# Non-Invasive Muscular Atrophy Causes Evaluation for Limb Fracture Based on Flexible Surface Electromyography System

**DOI:** 10.3390/s22072640

**Published:** 2022-03-30

**Authors:** Xiachuan Pei, Ruijian Yan, Guangyao Jiang, Tianyu Qi, Hao Jin, Shurong Dong, Gang Feng

**Affiliations:** 1 Key Laboratory of Advanced Micro/Nano Electronic Devices & Smart Systems of Zhejiang, College of Information Science and Electronic Engineering, Zhejiang University, Hangzhou 310027, China; 11731009@zju.edu.cn (X.P.); hjin@zju.edu.cn (H.J.); dongshurong@zju.edu.cn (S.D.); 2 Department of Orthopedic Surgery, 2nd Affiliated Hospital, School of Medicine, Zhejiang University, Hangzhou 310009, China; 11718243@zju.edu.cn (G.J.); 3130102372@zju.edu.cn (T.Q.); gangfeng@zju.edu.cn (G.F.); 3 International Campus, Zhejiang University, Haining 314400, China; 4 Dr. Li Dak Sum & Yip Yio Chin Center for Stem Cell and Regenerative Medicine, Zhejiang University, Hangzhou 310058, China

**Keywords:** flexible system, machine learning, muscular atrophy, surface electromyography

## Abstract

Muscular atrophy after limb fracture is a frequently occurring complication with multiple causes. Different treatments and targeted rehabilitation procedures should be carried out based on the causes. However, bedside evaluation methods are invasive in clinical practice nowadays, lacking reliable non-invasive methods. In this study, we propose a non-invasive flexible surface electromyography system with machine learning algorithms to distinguish nerve-injury and limb immobilization-related atrophy. First, a flexible surface electromyography sensor was designed and verified by in vitro tests for its robustness and flexibility. Then, in vivo tests on rats proved the reliability compared with the traditional invasive diagnosis method. Finally, this system was applied for the diagnosis of muscular atrophy in 10 patients. The flexible surface electromyography sensor can achieve a max strain of 12.0%, which ensures close contact with the skin. The in vivo tests on rats show great comparability with the traditional invasive diagnosis method. It can achieve a high specificity of 95.28% and sensitivity of 98.98%. Application on patients reaches a relatively high specificity of 89.44% and sensitivity of 91.94%. The proposed painless surface electromyography system can be an easy and accurate supplementary for bedside muscular atrophy causes evaluation, holding excellent contact with the body.

## 1. Introduction

Muscle atrophy caused by unloading or decreasing neural activity is often classified as disuse and commonly seen in patients with limb immobilization and peripheral nerve injury due to fracture [1,2,3]. Although both limb immobilization and peripheral nerve injury eventually lead to muscular dysfunction, their treatment and rehabilitation are different [4]. In the case of limb casting, muscular exercise after the release of immobilization can effectively restore muscle function. However, for patients with complicated nerve injury, rehabilitation of the nerve and muscle should be carried out simultaneously. Therefore, distinguishing between limb immobilization and peripheral nerve injury is important. Although several methods of the diagnosis of peripheral nerve injuries in clinical practices can provide quite promising results, such as magnetic resonance imaging, ultrasonic inspection, etc. [5], an alternative way of carrying out bedside diagnosis can be a great supplementary.

Electrophysiological studies, including needle electromyography (nEMG), is a commonly accepted bedside method to determine and quantify the function and disorders of the peripheral nervous system, specifically of the motor nerves and motor unit [6,7,8,9,10]. However, nEMG is invasive with pain during muscle activation, and prolonged recording is not possible [11]. Surface electromyography (sEMG) can easily solve the shortcomings of nEMG, while its accuracy and reliability remain a problem. The sEMG electrodes can hardly fit with the human body, which makes the results unconvincing. The low accuracy caused by focusing on time-domain or frequency-domain features only and the unappropriated analysis method also adds to the difficulty of applying it clinically. 

Recently, several researchers have focused on sEMG feature analysis to improve the performance of sEMG in clinical applications. Time-domain features have been utilized in diagnosing spinal and bulbar muscular atrophy, paediatric neuromuscular disorders, Parkinson’s disease, Amyotrophic lateral sclerosis (ALS) disease, etc. [11,12,13,14,15]. While the shortcomings still exist in the requirement of a constant contraction level and a stable output of distinctive single MU. As for the frequency-domain features, features such as Median Frequency (MDF), Mean Power Frequency (MPF), Zero-crossings (ZC) has already been extracted while the results had not shown clearly distinctive trends [16,17,18,19]. N.S. Arikidis develops an inter-scale wavelet maximum (ISWM) based on the time-frequency analysis for characterizing the electromyogram (EMG) interference pattern to assist in the diagnosis of neuromuscular disease and highly significant differences in ISWM values have been found [20]. Most research on sEMG take time-domain and frequency-domain features to analyse the health problems; a comprehensive combination of the two domains of multiple features with a smart analysis method might be a great solution towards this specific area of distinguishing the causes of muscle atrophy. 

Meanwhile, the method of analysis also shares a similar contribution to the accuracy of atrophy diagnosis as feature extraction. Several researchers turn to machine learning as the analysis method of sEMG to improve the accuracy and several algorithms are adopted. Swaroop uses neural networks to classify myopathy and neuropathy patients [21]. Xu Zhang adopts machine learning to diagnose ALS patients [15]. Wei proposes a wearable smart sEMG recorder integrated gradient boosting decision tree (GBDT) based hand gesture recognition [22]. A. B. M. S. U. Doulah adopts the K-nearest neighbourhood (KNN) for the classification of neuromuscular diseases [23]. The literatures above adopted machine learning algorithms as an improvement in the analysis method to make sEMG a reliable tool in different symptom monitoring. However, the absence of application in muscle atrophy analysis leaves big space for improvement in this specific area. 

Therefore, in this paper, a non-invasive flexible sEMG system (FSES) with machine learning algorithms was proposed for the accurate bedside diagnosis of the causes of muscular atrophy evaluation. It distinguishes nerve-injury and limb immobilization-related atrophy for patients after bone fracture based on flexible sEMG sensors. The proposed system overcomes the shortcomings of the traditional invasive method of diagnosis and improves the accuracy with the combined feature selection and the analysis method.

## 2. Materials and Methods

### 2.1. System Overview

**Figure 1:**
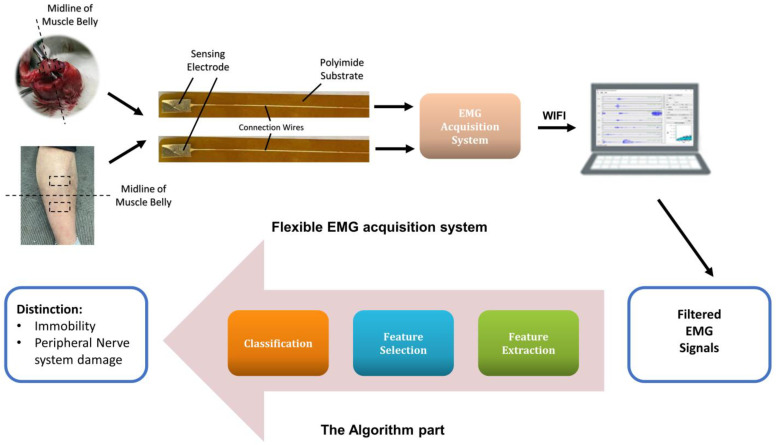
The block diagram of our flexible system.

As Figure 1 shows, the whole non-invasive system consists of the flexible EMG acquisition system and the algorithm part. The sEMG signals are acquired through the acquisition system while the subjects perform the activities designed. The raw signals captured by the laptop are filtered and then enter into the algorithm part. The final results present the accurate diagnosis between nerve-injury ones and limb immobilization ones for patients after bone fracture. The EMG acquisition and analysis are detailed explained in S1 (Supplementary Information). The raw data are sorted according to the channels and pass a 50 Hz notch filter to eliminate the noise in the environment, and then a fourth-order Butterworth filter of 20–500 Hz band-pass filter is applied to adjust the signal to the range of the electromyography frequency. The whole system has the robustness to deal with the noise.

### 2.2. Design and Fabrication of the Sensor

Details of principles and procedures of the fabrication process for the sensor part are mentioned in our previous paper [24]. Briefly, the sensor is covered by Polydimethylsiloxane (PDMS), with its conductive circuit part printed on the flexible polyimide (PI) substrate. The designed electrode part is glided with gold to improve conductivity and the size is set as a 0.9 cm × 0.4 cm rectangle, which we adjust to 4 cm × 4 cm in the later applications on patients. The whole sensor has excellent biocompatibility, great chemical stability, flexibility, and stretchability, which is quite crucial for in vivo tests to adjust to the variability of the complex circumstances.

### 2.3. In Vitro Tests

In vitro tests are carried out to test the stretchability and reliability of this system. The stretchability test is carried out by applying a uniaxial strain to both sides of the flexible system to explore the maximum length without breaking. The mechanical reliability test is performed by twisting the flexible system with an angle of 30° for hundreds of times. After each bend, the system’s SNR (signal/noise ratio) is evaluated by EMG signal acquisition.

### 2.4. In Vivo Tests

The clinical trials in this work, including tests on rats and patients, were approved by the Ethics Committee of the 2nd Affiliated Hospital, School of Medicine, Zhejiang University on 12 May 2020, and written consent was obtained from all the participants.

#### 2.4.1. Verification of the FSES System on Rats

##### Animals

Twelve Sprague Dawley rats aged 8 weeks and weighing 200–250 g were divided into two groups. The six rats in group 1 are the immobility group, which undertake days of limb suspension [25]; The other 6 rats in group 2 work as the nerve-injury group, of which the sciatic nerves are injured with scissors (*n* = 6).

##### Data Acquisition

**Figure 2:**
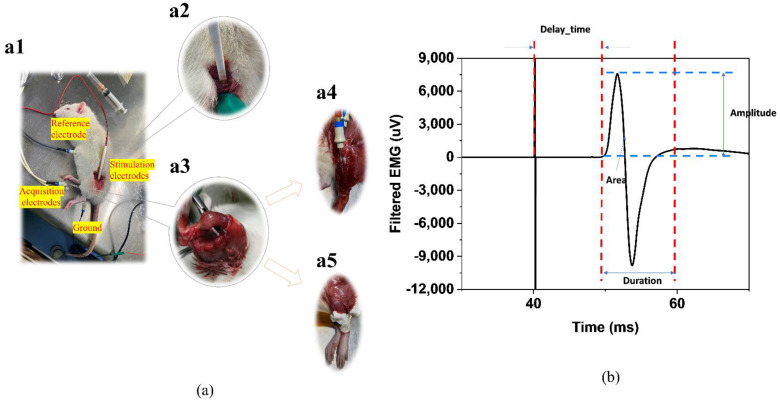
(**a**) (**a1**) The in vivo experiments on rats. (**a2**) The Hook-shaped stimulation electrodes. (**a3**) The tibialis anterior muscle of the rats on which we perform the EMG acquisition, including (**a4**) the concentric needle vs. (**a5**) the flexible surface electrode. (**b**) The representative traces of electrical stimulation-induced EMG wave and the parameters calculated.

In vivo tests of electromyography triggered by the electrical stimulus on rats are carried out to verify this flexible system. As Figure 2a shows, with stimulus electrodes placed on the sciatic nerves and acquisition electrodes placed on the muscle to be evaluated, the EMG signal will closely follow the stimulus artifact after performing the electrical stimulation each time [10]. Hook-shaped stimulation electrodes guarantee the contact and provide great protection of the nerves. After the removal of fur with hair removal cream and surface cleanse with 75% ethanol, the flexible sensors and the concentric needles are placed right onto the tibialis anterior muscle belly, respectively, on the upper and lower 0.5 cm from the mid-point of the muscle. A bipolar square wave (Amplitude: 2 mA, Frequency: 2 Hz, pulse width: 100 μs, duration time: 30 s) to rouse the slight movement of the lower limb of the rats is delivered by the stimulation electrodes to the intact node of the nerve and the triggered responses of the tibialis anterior muscle are acquired using the EMG system by the acquisition electrodes. nEMG was collected for the traditional invasive diagnosis and sEMG was collected for the verification of the system. Figure 2b shows the four common parameters used for calculation.

##### Feature Extraction

Several features have already been proposed in the analysis of sEMG. Root Mean Square (RMS), integrated EMG (iEMG), Average Rectified Value (ARV), etc. are widely used as time-domain features [16], and median frequency (MDF), mean power frequency (MPF) [16,26], entropy and recurrence quantification analysis (RQA), etc. are the ones often used in frequency-domain [13,14,27,28,29]. Several researchers have proved the strong relationship between RMS, iEMG and muscle strength, and MDF, MPF have also been proved to correlate with muscle fatigue. Muscle atrophy will affect the size and number of fibres, which leads to a decrease in the strength and the ability of anti-fatigue of the muscle. The adoption of these features can have a strong relationship with the situation of muscle atrophy. All the data are presented in the form of the ratio of the values of the affected subjects versus the values of the healthy subjects before the surgery.

##### Machine-Learning Algorithms

After feature extraction of EMG signals, these features can be used in the classification algorithm. Owing to the number of rats and the times of the patients rechecked, limited amount of data, sparse and stable distribution are the three main characteristics of the data. In this paper, three algorithms that suit the data were adopted and the hyperparameters were adjusted to achieve the best performance (Detailed descriptions in S2–S3).

##### Performance Metric for Classifier

Classifier performance is assessed based on three performance metrics which are accuracy, sensitivity, and specificity. (Details in S4) The test outcomes for any study can either be true positive (TP), true negative (TN), false positive (FP), or false negative (FN). The actual conditions were set into two classes, Positive and Negative, which means the immobility subjects and nerve-injury subjects in the model. The prediction can also be divided into two categories, True for the correct prediction and False for the wrong prediction, which means the accurate and inaccurate prediction of the patients.

(1) Accuracy=(TP+TN)/(TP+TN+FP+FN)

(2) Specificity=TN/(TN+FP) 

(3) Sensitivity=TP/(TP+FN)

#### 2.4.2. Clinical Applications

The flexible sensors were also applied to patients who suffer from bone fractures. All subjects gave informed consent to participate in this investigation after a full explanation of the study design and experimental procedures. The study was approved by the Second Affiliated Hospital of Zhejiang University. The number of limb immobilization subjects is seven, while the number of nerve-injury subjects is three. The medical conditions of the patients diagnosed by doctors and professional equipment are listed in Table 1 below.

At the time of examination, all patients were at hospital and the experiment was carried bedside. Four couples of differential electrodes are utilized (tibialis anterior muscle of the affected limb, gastrocnemius muscle of the affected limb, tibialis anterior muscle of the unaffected limb, gastrocnemius muscle of the unaffected limb). The size of the sensing electrode is adjusted to 4 × 4 cm to cover the muscle belly to collect EMG signal. After surface cleaning with 75% ethanol, each couple of electrodes are placed right in the muscle belly, respectively on the upper and lower 2 cm from the mid-point of the muscle. The patients are asked to perform the moves of flexion and extension of the ankle at their maximum voluntary contraction for 60 s during the signals acquisition. The protocol of the moves includes the extension and flexion of the ankle. All the data are presented in the form of the ratio of the affected limb versus the unaffected limb, which eliminates the influence of the patients’ different willingness to perform muscle contraction.

#### 2.4.3. Statistical Analysis

Continuous data are expressed as mean ± standard deviation (SD) and analysed by unpaired samples t-test between two groups. The star (*) indicates the level of statistical significance (*p*) < 0.05 tested at a 2-tailed. All data statistics were analysed by SPSS (Version 12.5).

## 3. Results

### 3.1. Flexibility and Reliability of FSES System

The elasticity, mechanical robustness of the flow sensor under various deformations, and harsh working environments such as clinical circumstances are essential requirements for flexible sensor systems. The maximum length of the serpentine part without breaking is 11.20 cm, while the original length is only 10.00 cm. 

The reliability of the sensor was also investigated. After each bending of the system, the ratio of contraction potential versus the resting potential of the EMG signal acquired by the flexible sensor is tested on the forearm with the same muscle strength. The flexible sensor was still working normally even after 500 cycles of an obvious bending (30°). 

### 3.2. In Vivo Tests on Rats 

#### 3.2.1. Traditional Diagnosis

**Figure 3:**
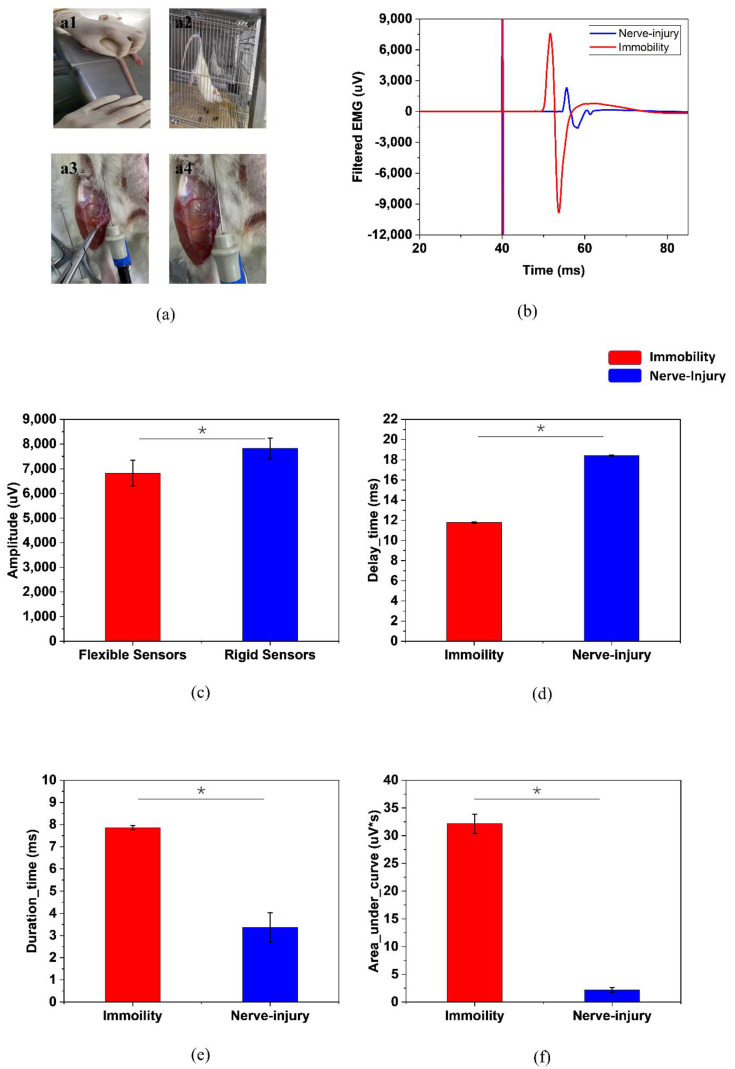
(**a**) Different groups of rats, (**a1**,**a2**) are the limb immobilization group while (**a3**,**a4**) are the nerve-injury group. (**b**) The distinct EMG responses between the two groups of rats. The comparations of (**c**) amplitude, (**d**) delay, (**e**) duration time (**f**) area under the curve between them. (* *p* < 0.05).

As Figure 3a,b shows, traditional nEMG diagnosis combined with the invasive inspection of the rats has been proved to be a quite accurate diagnosis method in experiments. Figure 3c–e shows the results of the comparison of nEMG features. 

#### 3.2.2. Statistical Features for Nerve-Injury and Limb Immobilization Rats’ EMG Signals

**Figure 4:**
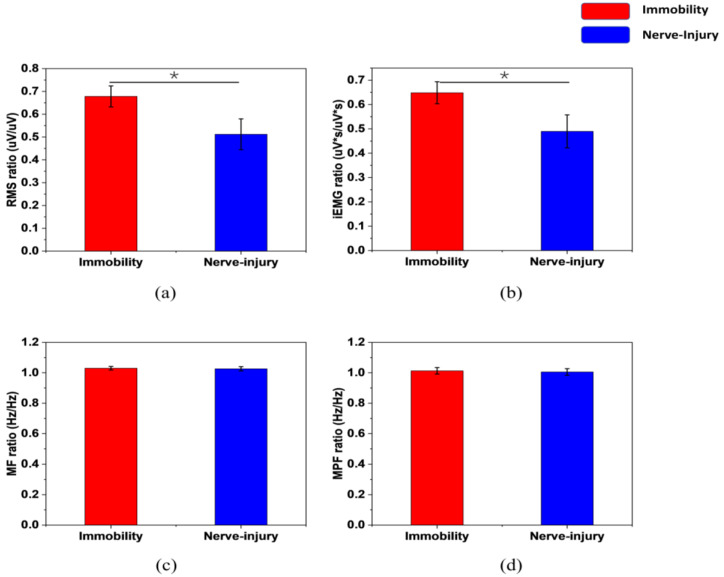
The sEMG features distribution of (**a**) RMS, (**b**) iEMG, (**c**) MF, (**d**) MPF between two categories. (* *p* < 0.05).

The features of the sEMG signals are shown in Figure 4, in the form of the ratio of the values of the affected subjects versus the values of the healthy subjects before the surgery. The data come from the average values of one limb immobilization rat and one nerve-injury rat, including 47 samples in total. 

#### 3.2.3. Classification with Features

The hyperparameters of each algorithm were adjusted to ensure the system had the best performance in distinguishing (more details in S3). The classification performance is listed in Table 2 below. In total, 80% of EMG signals were used as the training set and 20% of them were used as the test set, where the data from the training and test sets are randomly selected. The performance evaluation involved a 5-fold cross-validation that was repeated multiple times with each subject being, respectively, used for testing. The data includes 1800 samples in total as 150 times of data acquisition were performed on each rat, 900 of which is positive and 900 is negative.

### 3.3. Clinical Application on Patients

#### 3.3.1. Statistical Features for Nerve-Injury and Limb Immobilization Patients’ EMG Signals

**Figure 5:**
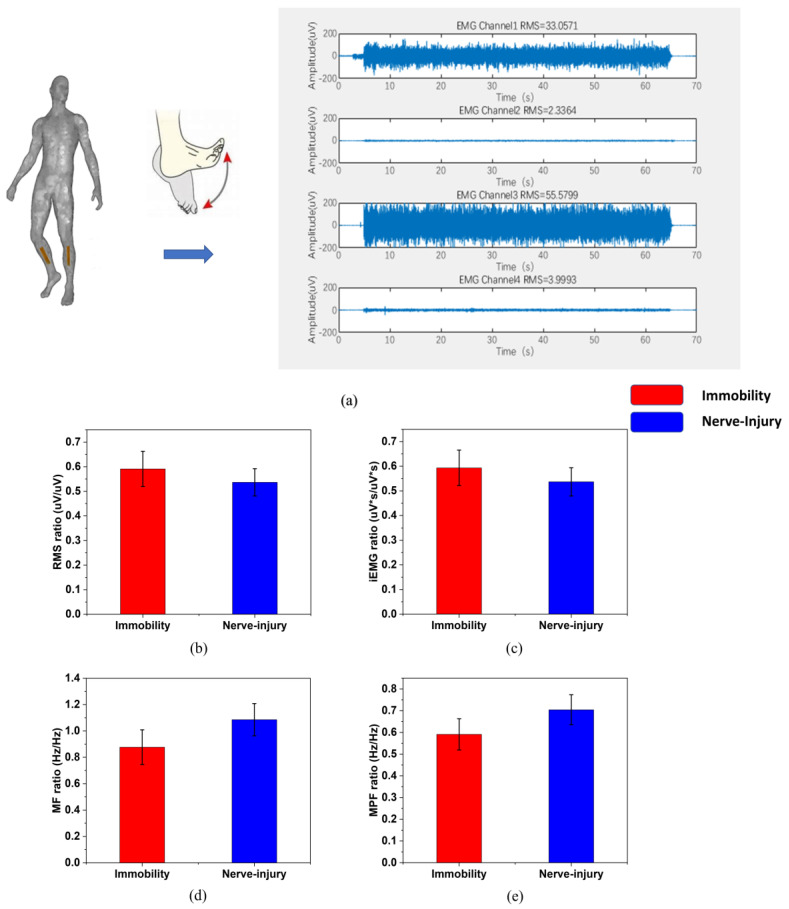
(**a**) The protocol of the moves and the data acquisition on the affected and unaffected sides. (The four channels are: tibialis anterior muscle of the affected limb, gastrocnemius muscle of the affected limb, tibialis anterior muscle of the unaffected limb, gastrocnemius muscle of the unaffected limb). The sEMG features are gathered every 0.5 s with an overlap of 1/8 after choosing the stable 30 s-period or 40 s-period from the 60 s-move. The distribution of (**b**) RMS, (**c**) iEMG, (**d**) MF, (**e**) MPF between nerve injury and limb immobilization patients after bone fracture.

The features extracted from one muscle-injury patient and one nerve-injury patient are shown in Figure 5 in the form of the ratio of the affected limb versus the unaffected limb (473 samples in total). 

#### 3.3.2. Classification with Features

The same features as the in vivo experiments on rats were adopted. We used the entire EMG signal fragments collected as the full sample, 80% of which were used as the training set and 20% of which were used as the test set, where the data from the training and test sets were randomly selected. The data includes 38,400 samples in total, 27,520 of which is positive and 10,800 is negative. The performance evaluation also involved 5-fold cross-validation.

As Table 3 shows, the accuracy of the two-tag label prediction can be observed. 

## 4. Discussion

In this study, a non-invasive flexible surface electromyography system with machine learning algorithms was proposed to distinguish nerve-injury and limb immobilization related atrophy. 

The system shows great stretchability. Despite the fact that the sensor contains circuit-scale components, the whole system can stand a large deformation (12.0%). This good stretchability is achieved with the flexible substrate, serpentine metal interconnects, and the PDMS cover layer. It exceeds the usual strain of human skin (30%), demonstrating a comfortable attachment for a human being. The reliability tests show that the ratio of contraction potential versus the resting potential of the EMG signal acquired by the flexible sensor stay stable during the whole bending process. It indicates that the system is robust and stable in harsh environments.

In traditional nEMG analysis, as Figure 2 shows, the distinct differences of the features combined with the invasive inspection of the rats make the diagnosis easy. The nerve-injury ones have a smaller amplitude, a shorter duration time, and a smaller area under the curve, while the latency between the electrical stimulation signal and the EMG signal increases. The peripheral nerve injury affects both the transmission from the nerve to the muscle and the number of muscle fibres activated to respond to the muscle contraction [6]. 

In sEMG analysis, as Figure 3 shows, both the time-domain and frequency-domain features of the limb immobilization ones have a large overlap with the ones of the nerve-injury ones. They do not have distinctive values between the two groups, while the FSES system achieves a high specificity of 95.28% and sensitivity of 98.98%. (Table 2) The evaluation of the muscle atrophy causes can be successfully achieved by the FSES system. It overcomes the shortcomings of the traditional invasive method of diagnosis and improves the accuracy with the combined feature extraction and adoption of the machine learning method.

After verification on rats, the FSES system was also applied to the diagnosis of patients. Although a slight decrease in accuracy was shown compared with the in vivo tests on rats, the FSES system achieved a relatively high specificity of 89.44% and sensitivity of 91.94%. (Table 3) It can be a great supplementary of bedside muscular atrophy evaluation of the causes, holding excellent contact with the body. Although several methods of the diagnosis of peripheral nerve injuries in clinical practices can provide quite promising results, such as magnetic resonance imaging, ultrasonic inspection, etc., reliable and accurate bedside diagnosis relies greatly on the traditional invasive nEMG. Our non-invasive flexible sEMG system with machine learning algorithms for the accurate bedside diagnosis of the causes of muscular atrophy evaluation can be a great alternative. It distinguishes nerve-injury and limb immobilization-related atrophy for patients after bone fracture based on flexible sEMG sensors and presents a quite promising result. The proposed system overcomes the shortcomings of the traditional invasive method of diagnosis. Additionally, it also improves the accuracy with the combined feature selection and the analysis method of machine learning algorithms. Potential values in clinical applications and further targeted rehabilitation procedures at home can be carried out based on the high accuracy of prediction of the system. 

The limitations of this study are the small sample size and the case–control study design. Future studies could consider larger sample sizes, evaluate men and women separately, or improve the classification accuracy.

## Figures and Tables

**Table 1:** Summary of the patients taken into consideration.

Patient No.	Fracture Diagnosis	Tags (Nerve-Injury = 0, Immobility = 1)	Gender
1	Tibial plateau fracture	1	Male
2	Tibial plateau fracture	0	Male
3	Patella fracture	1	Female
4	Radial head fracture	0	Female
5	Patella fracture	1	Female
6	Rehabilitation failure of elbow fracture	0	Female
7	Tibial fracture	1	Female
8	Tibial fracture	1	Male
9	Distal radius fracture	1	Male
10	Tibial fracture	1	Male

**Table 2:** Performance Comparison.

Classifiers	Accuracy (%)	Specificity (%)	Sensitivity (%)
XGBoost	96.67	95.28	98.98
SVM	95.56	95.77	95.56
KNN	95.78	94.22	97.72

**Table 3:** Performance Comparison.

Classifiers	Accuracy (%)	Specificity (%)	Sensitivity (%)
XGBoost	86.74	89.72	91.94
SVM	85.99	87.38	85.99
KNN	86.22	89.37	91.56

## Data Availability

The data presented in this study are available on request from the corresponding author.

## Supplementary Materials

The following supporting information can be downloaded at: https://www.mdpi.com/article/10.3390/s22072640/s1, supplementary information (S1–S4). S1. Detailed Explanation of the EMG Acquisition & Analysis; S2. Detailed Explanation of the Three Algorithms; S3. & Table S1. The Final Choice of the Hyperparameters; S4. Criteria of Classifiers’ Performance. References [30,31,32] are cited in the supplementary materials.
