# Supplementary material for: Non-Invasive Muscular Atrophy Causes Evaluation for Limb Fracture Based on Flexible Surface Electromyography System
Source: Sensors (Basel). 2022 Mar 30;22(7):2640. doi: 10.3390/s22072640 (PMC9003361; doi:10.3390/s22072640)
Supplement: Supplementary file 1 [file sensors-22-02640-s001.zip › sensors-1620515-supplementary.pdf]

sensors

MDPI

Supplementary Information

Non-Invasive Muscular Atrophy Causes Evaluation for Limb Fracture Based on Flexible Surface Electromyography System

Xiachuan Pei [1,2], Ruijian Yan [2,*], Guangyao Jiang [2], Tianyu Qi [2], Hao Jin [1,3,4], Shurong Dong [1,3] and Gang Feng [2]

1 Key Laboratory of Advanced Micro/Nano Electronic Devices & Smart Systems of Zhejiang, College of Information Science and Electronic Engineering, Zhejiang University, Hangzhou 310027, China; 11731009@zju.edu.cn (X.P.); hjin@zju.edu.cn (H.J.); dongshurong@zju.edu.cn (S.D.)
2 Department of Orthopedic Surgery, 2nd Affiliated Hospital, School of Medicine, Zhejiang University, Zhejiang 310009, China; 11718243@zju.edu.cn (G.J.); 3130102372@zju.edu.cn (T.Q.); gangfeng@zju.edu.cn (G.F.)
3 International Campus, Zhejiang University, Haining 314400, China
4 Dr. Li Dak Sum & Yip Yio Chin Center for Stem Cell and Regenerative Medicine, Zhejiang University, Hangzhou 310027, China
* Correspondence: yanruijian@zju.edu.cn

Citation: Pei, X.; Yan, R.; Jiang, G.; Qi, T.; Jin, H.; Dong, S.; Feng, G. Non-Invasive Muscular Atrophy Causes Evaluation for Limb Fracture Based on Flexible Surface Electromyography System. *Sensors* **2022**, *22*, 2640. https://doi.org/10.3390/s22072640

Academic Editor: Seokheun Choi

Received: 16 February 2022
Accepted: 23 March 2022
Published: 30 March 2022

Publisher's Note: MDPI stays neutral with regard to jurisdictional claims in published maps and institutional affiliations.

Copyright: © 2022 by the authors. Submitted for possible open access publication under the terms and conditions of the Creative Commons Attribution (CC BY) license (https://creativecommons.org/licenses/by/4.0/).

S1. EMG Acquisition & Analysis

We use the ADS1299 biopotential measurement chip, which includes four Analog-to-digital converter (ADC) chips with 24-bit resolution, high input impedance, and low input noise, as the core chips of our data acquisition tool EMG32. Each channel is composed of two differential electrodes. A custom software is developed to accurately determine which of the two electrodes in each channel has fallen off which means we can obtain the best assessment effect by adjusting the position, increasing the intensity of the pressure, or other operations in real-time. With all 4 channels turned on, the sampling rate could reach 16k Samples/s per channel. We manage the data files on our laptop later. The raw data get sorted according to the channels and pass a 50Hz notch filter to eliminate the noise in the environment, and then a fourth-order Butterworth filter of 20–500 Hz band-pass filter is applied to adjust the signal to the range of the electromyography frequency.

S2. Detailed Explanation of the Three Algorithms

Support vector machine (SVM) is a method of creating nonlinear classifiers by applying the kernel trick to the maximum-margin hyperplane [30]. This classification method is good for processing with small data sets, but large characteristic dimensions. It is effective even if there are lots of noises in the data. This characteristic perfectly suits our limited amount of data.

We also adopt the eXtreme Gradient Boosting algorithm (XGBoost) to help our classification. XGBoost is an optimization of GBDT (Gradient Boosting Decision Tree). It is highly efficient, flexible and portable, and is widely used in data mining, recommendation systems, and other fields [31]. It considers the special condition of sparse training data, you can specify the default direction of the branch for the missing value or the specified value.

K Nearest Neighbors algorithm (KNN) is a non-parametric pattern recognition method proposed by Thomas Cover and has been used for classification and regression [32]. Generally, the class of a sample is determined by the mode of the class of k adjacent samples. KNN is easy to understand and has a fast speed in the training process. When the data are stable and have few outliers, KNN can be quite suitable. It is suitable when the amount of data is small.

S3. The Final Choice of the Hyperparameters

The table below shows the final choice of the hyperparameters.

Table S1. Hyperparameters Of the Algorithm.

Classifiers	Hyperparameters
XGBoost	Booster = gbtree,<br>Objective = multi: softprob,<br>n_estimators = 100,<br>max_depth = 6,<br>min_child_weight = 1,<br>gamma = 0,<br>learning_rate = 0.3.
SVM	C = 10, gamma = 0.001.
KNN	K = 1, P =12

S4. Criteria of Classifiers' Performance

The figure below shows the test outcomes for any study can either be true positive (TP), true negative (TN), false positive (FP), or false negative (FN), based on the real condition and the prediction.

**True Positive (TP):**<br>• Reality: Immobility<br>• Prediction: Immobility<br>• Outcome: Hit	**False Positive (FP):**<br>• Reality: Nerve-injury<br>• Prediction: Immobility<br>• Outcome: Miss
**False Negative (FN)::**<br>• Reality: Immobility<br>• Prediction: Nerve-injury<br>• Outcome: Miss	**True Negative (TN):**<br>• Reality: Nerve-injury<br>• Prediction: Nerve-injury<br>• Outcome: Hit

Figure S1. The four possible results of the prediction.
